# Loss of TBC1D2B causes a progressive neurological disorder with gingival overgrowth

**DOI:** 10.1038/s41431-024-01563-5

**Published:** 2024-02-19

**Authors:** Frederike L. Harms, Jessica Erin Rexach, Stephanie Efthymiou, Busra Aynekin, Hüseyin Per, Ayten Güleç, Sheela Nampoothiri, Hugo Sampaio, Rani Sachdev, Radka Stoeva, Kasiani Myers, Loren D. M. Pena, Theodosia A. Kalfa, Marisa Chard, Megan Klassen, Megan Pries, Kerstin Kutsche

**Affiliations:** 1 Institute of Human Genetics, University Medical Center Hamburg‐Eppendorf, Hamburg, Germany; 2 Department of Neurology, Program in Neurogenetics, David Geffen School of Medicine, University of California Los Angeles, Los Angeles, CA USA; 3 Department of Neuromuscular Disorders, UCL Queen Square Institute of Neurology, London, WC1N 3BG UK; 4 Division of Pediatric Neurology, Department of Pediatrics, Faculty of Medicine, Erciyes University, Kayseri, Turkey; 5 Department of Pediatric Genetics, Amrita Institute of Medical Sciences and Research Centre, Cochin, Kerala India; 6 Department of Women and Children’s Health, University of New South Wales, Randwick Campus, Randwick, NSW Australia; 7 Sydney Children’s Hospital, Randwick, NSW Australia; 8 Centre for Clinical Genetics, Sydney Children’s Hospital, Randwick, NSW Australia; 9 School of Women’s and Children’s Health, University of New South Wales, Randwick, NSW Australia; 10 Department of Medical Genetics, Le Mans Hospital, Le Mans, France; 11 Division of Bone Marrow Transplant, Cincinnati Children’s Hospital Medical Center, Cincinnati, OH USA; 12 Department of Pediatrics, University of Cincinnati College of Medicine, Cincinnati, OH USA; 13 Division of Human Genetics, Cincinnati Children’s Hospital Medical Center, Cincinnati, OH USA; 14 Division of Hematology, Cincinnati Children’s Hospital Medical Center, Cincinnati, OH USA; 15 Provincial Medical Genetics Program, Newfoundland and Labrador Health Services, St. John’s, NL Canada; 16 Department of Pediatrics, Memorial University Faculty of Medicine, St. John’s, NL Canada

**Keywords:** Genetics research, Experimental models of disease

## Abstract

Biallelic loss-of-function variants in *TBC1D2B* have been reported in five subjects with cognitive impairment and seizures with or without gingival overgrowth. TBC1D2B belongs to the family of Tre2-Bub2-Cdc16 (TBC)-domain containing RAB-specific GTPase activating proteins (TBC/RABGAPs). Here, we report five new subjects with biallelic *TBC1D2B* variants, including two siblings, and delineate the molecular and clinical features in the ten subjects known to date. One of the newly reported subjects was compound heterozygous for the *TBC1D2B* variants c.2584C>T; p.(Arg862Cys) and c.2758C>T; p.(Arg920*). In subject-derived fibroblasts, *TBC1D2B* mRNA level was similar to control cells, while the TBC1D2B protein amount was reduced by about half. In one of two siblings with a novel c.360+1G>T splice site variant, *TBC1D2B* transcript analysis revealed aberrantly spliced mRNAs and a drastically reduced *TBC1D2B* mRNA level in leukocytes. The molecular spectrum included 12 different *TBC1D2B* variants: seven nonsense, three frameshifts, one splice site, and one missense variant. Out of ten subjects, three had fibrous dysplasia of the mandible, two of which were diagnosed as cherubism. Most subjects developed gingival overgrowth. Half of the subjects had developmental delay. Seizures occurred in 80% of the subjects. Six subjects showed a progressive disease with mental deterioration. Brain imaging revealed cerebral and/or cerebellar atrophy with or without lateral ventricle dilatation. The *TBC1D2B* disorder is a progressive neurological disease with gingival overgrowth and abnormal mandible morphology. As TBC1D2B has been shown to positively regulate autophagy, defects in autophagy and the endolysosomal system could be associated with neuronal dysfunction and the neurodegenerative disease in the affected individuals.

## Introduction

Biallelic loss-of-function variants in *TBC1D2B* have previously been linked with a complex and evolving phenotype in three unrelated patients [1]. In the initial three cases, a characteristic phenotypic pattern was evident, encompassing neurodevelopmental and neurodegenerative traits, along with distinctive features of facial and gingival overgrowth. Specifically, a boy and a girl presented with mild developmental delay and mild intellectual disability, respectively. The third subject developed normally until 12–13 years of age and then showed mental deterioration that was accompanied by slurred speech and gait ataxia. In late adolescence, he developed visual deterioration, flexion contractures, and limb tremor. Seizures occurred in all subjects. Gingival overgrowth became apparent in early childhood. Cherubism was identified in the girl through the presence of enlargement of upper and lower jaws and multilocular, radiolucent lesions of the mandible in the orthopantogram [1]. Subsequently, two adult brothers carrying a homozygous *TBC1D2B* 1-bp deletion were reported with a similar clinical course [2].

*TBC1D2B* encodes a member of the family of Tre2-Bub2-Cdc16 (TBC)-domain containing RAB-specific GTPase activating proteins (TBC/RABGAPs) that are key regulators of RAB proteins [3, 4]. Members of the family of RAB small GTPases switch between an active, GTP-bound and an inactive, GDP-bound state and are required for endolysosomal membrane trafficking [5]. TBC1D2B binds various RAB proteins, including RAB31, and can transform active RAB proteins into their inactive forms through its GTPase activating domain [6–8]. In HeLa cells, TBC1D2B was found to co-localize with vesicles positive for RAB5 and partially with EEA1, both markers of early endosomes [1]. To date, three nonsense and three early frameshift variants of *TBC1D2B* were reported in affected individuals [1, 2]. In subject fibroblasts with the homozygous p.(Leu793*) variant and the compound heterozygous variants p.(Leu220Glufs*6) and p.(Tyr765*), *TBC1D2B* mRNA levels were drastically reduced to 10–20% compared to control cells and the TBC1D2B protein was absent. In a *TBC1D2B* cellular knockout model, reduced epidermal growth factor internalization and enhanced susceptibility to cell death under serum starvation condition were detected. The data led us to suggest that combined defects in vesicle trafficking and cell survival may underlie the neurodegenerative phenotype in subjects with TBC1D2B deficiency [1].

Here we report on five new subjects with novel biallelic *TBC1D2B* pathogenic variants. We investigated the impact of *TBC1D2B* variants on transcript and protein levels in leukocytes or fibroblasts from two subjects. Our findings support the loss-of-function nature of *TBC1D2B* variants. To describe the clinical spectrum of loss of TBC1D2B, we report genetic variants and detailed clinical phenotypic data of 10 total cases, including five newly reported cases, two brothers with a homozygous *TBC1D2B* frameshift variant [2], and the updated phenotypic data from the original cohort of three subjects with homozygous or compound heterozygous *TBC1D2B* variants [1]. Our findings define the core phenotypic traits associated with *TBC1D2B* loss-of-function, establishing a distinct and recognizable genetic syndrome.

## Subjects and methods

### Subjects

Informed consent for genetic analyses was obtained for subjects 7–11. Genetic studies were performed clinically or as approved by local Institutional Review Boards at University College London Hospitals (London, United Kingdom), Erciyes University (Kayseri, Turkey), Cincinnati Children’s Hospital (Cincinnati, USA), and Newfoundland and Labrador Health Services (Newfoundland and Labrador, Canada). The parents of the subjects provided written informed consent for participation in the study, clinical data and specimen collection, genetic analysis, and publication of relevant findings. Written informed consent for the publication of photographs was obtained for five subjects. Subject 10 and parents were enrolled in the Congenital Dyserythropoietic Anemia (CDA) Registry of North America (CDAR; clinicaltrials.gov NCT02964494), since the patient was diagnosed with pancytopenia and dyserythropoiesis.

### Exome or genome sequencing and variant validation and segregation

Genomic DNA was extracted from peripheral blood samples using standard procedures. Sequencing was performed by either trio exome sequencing (subjects 7 and 11 with their respective parents), trio whole genome sequencing (subject 10 and parents), or proband-only exome sequencing (subject 8) (Supplementary information). When necessary, *TBC1D2B* variants were validated and segregated by Sanger-sequencing using either fibroblast-derived DNA (subject 7) or leukocyte-derived DNA (subjects 8 and 9 and parents) (Supplementary information). Primer sequences can be found in Supplementary Table 1.

### Cell culture of primary dermal fibroblasts

Primary fibroblasts were cultured as described (Supplementary information) [1].

### RNA isolation, cDNA synthesis, RT-PCR and Sanger-sequencing, and quantitative real-time PCR (RT-qPCR)

To analyze *TBC1D2B* transcripts, RNA isolation from fibroblasts, complementary DNA (cDNA) synthesis, reverse transcription polymerase chain reaction (RT-PCR), and Sanger-sequencing of amplicons to analyze *TBC1D2B* transcripts were performed as described [9]. RT-qPCR was performed to determine the relative mRNA levels of *TBC1D2B* as described (Supplementary information) [9]. Primer sequences can be found in Supplementary Table 1.

### Immunoblotting

Whole-cell lysates from subject and control fibroblasts were prepared and immunoblotting was performed as described (Supplementary information) [1]. The antibodies used are described in Supplementary information.

### Data analysis and statistics

Quantitative data are presented by GraphPad Prism 8 software (Instat, GraphPad Software) as the mean ± standard deviation (SD). For quantification, one- or two-way ANOVA followed by a Dunnett *post hoc* test for multiple comparisons was performed. A *P* < 0.05 was considered statistically significant (**P ≤ 0.01; ***P ≤ 0.001).

## Results

### Molecular genetic investigations

Since the first report on *TBC1D2B* as a novel disease gene for an autosomal recessive neurological disorder, we recruited five new patients (subjects 7 to 11) with biallelic *TBC1D2B* variants through GeneMatcher [10] or referral, including three unrelated subjects (subjects 7, 10, and 11) and a female patient and her brother (subjects 8 and 9) (Table 1).

**Table 1:** Clinical characteristics of previously published and new subjects with biallelic *TBC1D2B* variants.

	Follow-up data of subjects published by Harms et al. [1]			Correia-Costa et al. [2]		This study					total
	Family 1	Family 2	Family 3	Family 4		Family 5	Family 6		Family 7	Family 8	
	Subject 1	Subject 3	Subject 4	Subject 5	Subject 6	Subject 7	Subject 8	Subject 9	Subject 10	Subject 11	
General information											
Ethnicity	Indian	European, Portuguese	European, Chechen	Latin, Brazilian		European, Norwegian/ Polish	Turkish		European-American (of German ancestry)	European (English/Irish ancestry)	
Sex	male	female	male	male	male	female	female	male	female	female	
Age at last examination	Follow-up at 31 y	Follow-up at 12.5 y	Follow-up at 3.5 y	36 y	25 y	25 y	18 y	29 y	10 y	35 y	
Alive/Dead	alive	alive	alive	died at 39 y	alive	died at 26 y 10 m	died at 21 y	alive	alive	alive	
*TBC1D2B* variant (NM_144572.2/ NP_653173.1)	homozygous c.2378T>A; p.(Leu793*)	compound heterozygous c.426dup; p.(Asn143*), c.1480C>T; p.(Gln494*)	compound heterozygous c.658_659del; p.(Leu220Glufs*6), c.1480C>T; p.(Gln494*)	homozygous c.595del ; p.(Val199Trpfs*22)		compound heterozygous c.2584C>T; p.(Arg862Cys), c.2758C>T; p.(Arg920*)	homozygous c.360+1G>T		compound heterozygous c.159C>G; p.(Tyr53*), c.2353C>T; p.(Arg785*)	homozygous c.25G>T; p.(Glu9*)	
Consanguinity	─	─	─	+		─	+		─	─	
Abnormality of the face (HP:0000271)											
Coarse facial features (HP:0000280)	+	─	no data	+	+	─	+	+	─	─	5/9
Gingival overgrowth (HP:0000212)	+	+	+ (3 y)	+ (3 y)	+ (3 y)	+^a^ (18 m)	+	─	+ (early childhood)	─	8/10
Abnormal mandible morphology (HP:0000277)	+	+	─	+ (3 y)	+	+	+	+	+	─	8/10
Numerous pigmented freckles (HP:0007587)	+	─	─	─	─	+ (22 y)	─	─	─	─	2/10
Abnormality of the nervous system (HP:0000707)											
Global developmental delay (HP:0001263)	─	+	+	─	─	+	─	+	+	─	5/10
Mental deterioration (HP:0001268)	+	─	─	+ (15 y)	+ (15 y)	+ (20 y)	+ (10 y)	─	─	+ (5 y)	6/10
Slurred speech (HP:0001350)	+ severe	─	─ absent speech	+ (17 y)	+ (12 y)	─ staccato speech	+ (10 y)	─ absent speech	─ about 30 words	─	4/10
Gait disturbance (HP:0001288)	+ gait ataxia	─	+ inability to walk	+ gait ataxia at 17 y, loss of ambulation at 20 y, bedridden at 36 y	+ gait ataxia at 15 y, bedridden at 25 y	+ gait ataxia at 21 y, wheelchair-bound at 23.5 y	+ gait ataxia, bedridden at 12 y	─	+ unsteady gait	─	7/10
Abnormality of movement (HP:0100022)	+ hand tremor	not reported	not reported	+ limb tremor at 36 y	+ limb and intention tremor at 25 y	not reported	+ dystonia at 8 y	not reported	+ myoclonus	+ intermittent hand tremor since childhood	6/6
Seizures (HP:0001250)	─ controlled on medication	─ controlled on medication	─ controlled on medication	+ (32 y)	–	+ (9 m)	+ (2 y and 12 y)	─	+ (13 m)	+ (4 m, 34 y) controlled on medication by her teen years	8/10
EEG abnormality (HP:0002353)	─ no recent EEG	+ no recent EEG	+	not done	not done	─ (20 y)	+ (16 y)	─	+ (13 m)	+ (28 y)	5/8
Behavioral abnormality (HP:0000708)	+	─	─	─	─	+ (14 y)	+	+	─	+	5/10
CT brain abnormalities	+ (23 y) no recent CT	─ no recent CT	+ no recent CT	+ (36 y)	not done	not done	not done	not done	not done	+ (34 y)	4/5
Brain MRI abnormalities	+ (14 y) no recent MRI	─ (2 y) no recent MRI	+ (3 y)	not done	+ (11 y)	+ (18 y)	+ (10 y 6 m) normal at 10 y	not done	+ (18 m)	+ (34 y)	7/8
Eye and hearing abnormalities (HP:0000478; HP:0000364)											
Visual loss (HP:0000572)	+	─	+ (3 y)	not reported	─	+ (36 w) retinopathy of pre-maturity	+ (20 y)	+ (20 y)	─	─	5/9
Abnormal fundus morphology (HP:0001098)	+	+ unchanged	− (3 y)	not reported	not reported	+	not done	not done	─	not done	3/5
Abnormal retinal morphology on macular OCT (HP:0030612)	+ unchanged	─	not done	not reported	not reported	not done	not done	not done	not done	not done	1/2
Hearing impairment (HP:0000365)	+	─ (10.5 y)	+	no data	no data	− (25 y)	no data	no data	─	+	3/6
Additional abnormalities											
Flexion contracture (HP:0001371)	+	+	+ (3.5 y)	+ (36 y)	−	+	+ (12 y)	+ (4–5 y)	─	─	7/10
Respiratory failure requiring assisted ventilation (HP:0004887)	─	**─**	**─**	+ tracheostomy at 15 y, dependent on assisted ventilation at 36 y	+ tracheostomy at 15 y	─	+ tracheostomy at 14 y	─	─	─	3/10
Abnormality of blood and blood-forming tissues (HP:0001871)	not reported	not reported	not reported	not reported	+ idiopathic pancytopenia	+ thrombocytopenia	not reported	+ leukopenia	+ pancytopenia	+ mild lymphopenia and thrombocytopenia	5/5

Age of onset is given in brackets.

*+* present, *–*  absent, *CT* computerized tomography, *m* months, *MRI* magnetic resonance imaging, *OCT* optical coherence tomography, *w* weeks, *y* years.

^a^Possibly drug induced.

**Fig. 1:**
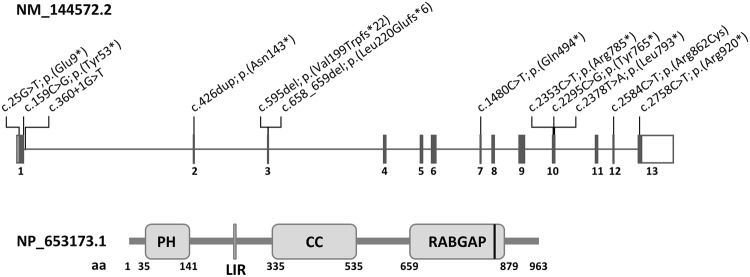
Schematics of the *TBC1D2B* gene and protein with locations of the pathogenic variants. Exon-intron structure of the *TBC1D2B* gene (top) showing the locations of the pathogenic variants. Exons are indicated by boxes and introns by lines. The 5′ untranslated region is depicted in light gray, the 3′ untranslated region in white and the coding region in dark gray. Exon numbering and variant description are given according to the mRNA reference number NM_144572.2. The TBC1D2B domain structure (bottom) with amino acids (aa) numbered according to NP_653173.1. The position of the amino acid residue arginine 862 substituted by cysteine [p.(Arg862Cys)] is highlighted by a black line. Domain assignment was determined using the Simple Modular Architecture Research Tool (SMART) server, available at http://smart.embl-heidelberg.de/. CC coiled coil domain, LIR LC3-interacting region (aa 252-EEWELLD-258), PH pleckstrin homology domain, RABGAP RABGTPase-activating protein domain.

Trio exome sequencing identified compound heterozygous *TBC1D2B* (NM_144572.2) variants c.2584C>T; p.(Arg862Cys) and c.2758C>T; p.(Arg920*) in subject 7 (Fig. 1, Table 1 and Supplementary Table 2). By single exome sequencing, the 18-year-old female subject 8 was found to carry the homozygous transition c.360+1G>T affecting the highly conserved splice donor site in intron 1 of *TBC1D2B* (Fig. 1, Table 1 and Supplementary Table 2). Segregation analysis revealed the same homozygous *TBC1D2B* splice site variant in her similarly affected brother (subject 9) (Table 1 and Supplementary Fig. 1) and both healthy parents were heterozygous carriers of the variant (Supplementary Fig. 1). Trio genome sequencing in subject 10 and parents detected compound heterozygous *TBC1D2B* nonsense variants c.159C>G; p.(Tyr53*) and c.2353C>T; p.(Arg785*) in subject 10 (Fig. 1, Table 1, Supplementary Fig. 2 and Supplementary Table 2). In subject 11, the homozygous *TBC1D2B* nonsense variant c.25G>T; p.(Glu9*) was identified by trio exome sequencing (Fig. 1, Table 1 and Supplementary Table 2). The *TBC1D2B* variants p.(Arg862Cys) and c.360+1G>T are absent in the gnomAD database (v4.0.0), while p.(Glu9*) has a minor allele frequency of 0.001198%, p.(Tyr53*) of 0.000522%, p.(Arg785*) of 0.000138%, and p.(Arg920*) of 0.000518% in the gnomAD database v4.0.0 (Supplementary Table 2). The four nonsense variants are likely loss-of-function alleles, but because p.(Arg920*) is located in the last exon of *TBC1D2B*, it potentially escapes nonsense-mediated mRNA decay (NMD). The missense variant c.2584C>T; p.(Arg862Cys) is predicted to have a deleterious impact on protein function by several in silico tools (Supplementary Table 2). Arginine 862 is located in the RABGAP domain (amino acids 659–879) of TBC1D2B (NP_653173.1; Fig. 1) and shows evolutionary conservation between species (Supplementary Fig. 3). According to the guidelines of the American College of Medical Genetics and Genomics and the Association for Molecular Pathology [11], we interpreted *TBC1D2B* variants c.25G>T; p.(Glu9*) (PVS1, PP4_strong, PM2_supporting, and PM3_supporting), c.159C>G; p.(Tyr53*), c.2353C>T; p.(Arg785*) (for both: PVS1, PP4_strong, PM2_supporting, and PM3), c.2758C>T/p.(Arg920*) (PVS1_moderate, PP4_strong, PM2_supporting, PM3, and PS3), and c.360+1G>T (PVS1_RNA, PM2_supporting, PM3, and PP4_strong) as pathogenic variants, whereas the missense variant c.2584C>T; p.(Arg862Cys) (PP4_strong, PP3_supporting, PM2_supporting, and PM3) was interpreted as likely pathogenic variant. We, therefore, believe that the biallelic *TBC1D2B* variants likely explain the phenotype in subjects 7 to 11.

### TBC1D2B transcript and protein analyses

**Fig. 2:**
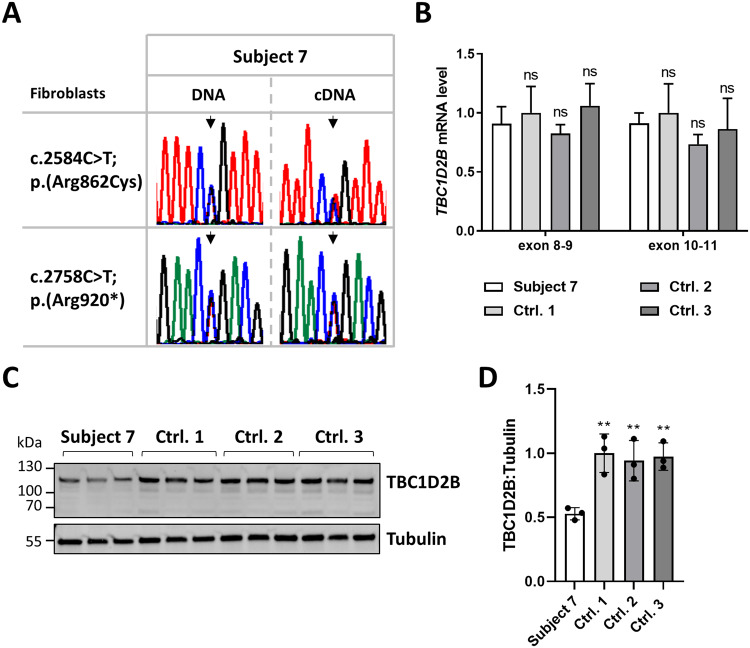
*TBC1D2B* transcript analysis and determination of TBC1D2B protein level in fibroblasts of subject 7. **A** Validation of the *TBC1D2B* c.2584C>T and c.2758C>T variants in DNA and transcript analysis using cDNA isolated from subject 7-derived fibroblasts. Partial sequence electropherograms showing the *TBC1D2B* c.2584C>T; p.(Arg862Cys) and c.2758C>T; p.(Arg920*) variants in fibroblast-derived DNA of subject 7 in the heterozygous state (left column). The right column shows sequence electropherograms of *TBC1D2B* transcripts obtained from fibroblast-derived cDNA of subject 7. The ratio of both mutant *TBC1D2B* transcripts is ~1:1. Arrows point to the position of the pathogenic variant. **B** Relative *TBC1D2B* mRNA levels determined by real-time quantitative PCR (RT-qPCR) using two primer pairs (exon 8–9 and exon 10–11) with fibroblast-derived cDNA of subject 7 and three controls normalized to *GAPDH* mRNA levels. The mean ± SD of one experiment performed in triplicate is shown. **C** Immunoblot analysis of lysates obtained from fibroblasts of subject 7 and three controls from three different passages. The amount of TBC1D2B was monitored by using an anti-TBC1D2B antibody. An anti-Tubulin antibody was used to control for equal loading. **D** Band intensities were quantified using a chemiluminescence imager. TBC1D2B was normalized relative to Tubulin. The mean ± SD of three independent experiments is shown. One-way ANOVA followed by Dunnett *post-hoc* test: ^**^P ≤ 0.01. cDNA complementary DNA, Ctrl. 1–3 fibroblast controls, ns not significant.

We used fibroblasts of subject 7 to analyze the effect of the *TBC1D2B* variants p.(Arg862Cys) and p.(Arg920*) on RNA and protein level. We first confirmed the presence of both *TBC1D2B* variants in genomic DNA isolated from subject 7-derived fibroblasts (Fig. 2A). Next, we used qualitative RT-PCR to amplify *TBC1D2B* transcripts harboring the missense variant c.2584C>T; p.(Arg862Cys) and the nonsense variant c.2758C>T; p.(Arg920*). Sequence analysis of the amplicons revealed a ~1:1-ratio of both *TBC1D2B* mutant transcripts suggesting NMD escape of *TBC1D2B* mRNAs with the premature stop codon in fibroblasts of subject 7 (Fig. 2A). This was confirmed by quantitative real-time PCR as we detected similar *TBC1D2B* mRNA levels in subject 7 and control fibroblasts by using two different primer sets (Fig. 2B). We next determined TBC1D2B protein levels by immunoblotting and detected a statistically significant reduced level by ~50% in subject 7 compared to control cells (Fig. 2C, D). A C-terminally truncated TBC1D2B protein [p.(Arg920*)] with a predicted molecular mass of ~105 kDa (compared to wild-type TBC1D2B with ~110 kDa) could not be detected in cells from subject 7 (Fig. 2C), although we used an anti-TBC1D2B antibody that recognizes the central amino acids 372–452 (epitope). These data suggest that the TBC1D2B-Arg862Cys mutant protein seems to be stable, while the TBC1D2B mutant with loss of the last 44 amino acid residues at the C-terminus [p.(Arg920*)] is potentially unstable and/or degraded in fibroblasts of subject 7.

**Fig. 3:**
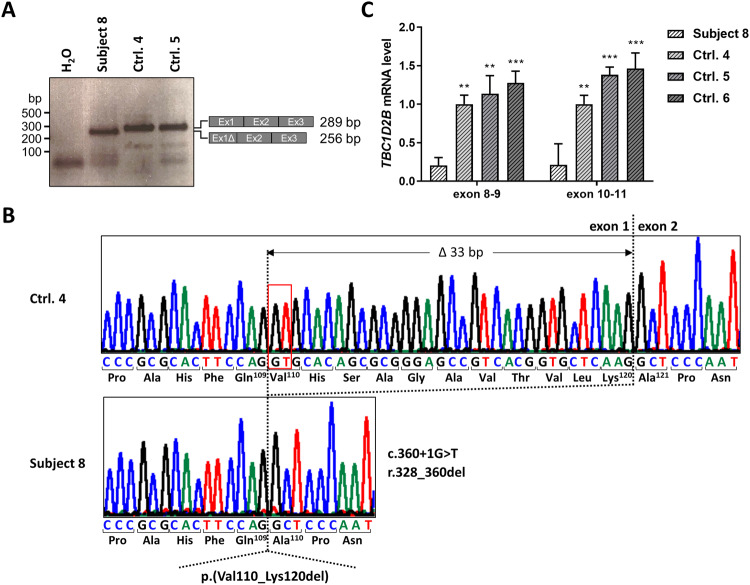
*TBC1D2B* transcript analysis of subject 8 leukocytes. **A** Agarose gel showing RT-PCR products with a primer pair for *TBC1D2B* exons 1–3 using leukocyte-derived cDNA of subject 8 and two controls. Schematics of the exon-exon junctions and the size of the amplicons (after sequencing) are depicted on the right. A single amplicon of ~250 bp was generated by RT-PCR in subject 8 and of ~290 bp in controls. **B** Partial sequence electropherograms obtained from Sanger-sequencing of RT-PCR amplicons of subject 8 and control 4. Exon numbering is given. Dotted lines mark the junction between exons 1 and 2. A cryptic splice donor site 33 bp upstream of the canonical splice donor site (red rectangle) is used in leukocytes of subject 8 due to the homozygous *TBC1D2B* c.360+1G>T intron 1 variant. The resulting *TBC1D2B* transcripts lack 33 bp of exon 1 (Δ33 bp; r.328_360del), which is predicted to cause an *in-frame* loss of 11 amino acids on protein level [p.(Val110_Lys120del)]. **C** Relative *TBC1D2B* mRNA levels determined by real-time quantitative PCR (RT-qPCR) using two primer pairs (exon 8–9 and exon 10–11) with leukocyte-derived cDNA of subject 8 and three controls normalized to *GAPDH* mRNA levels. Two independent RNA isolations (Experiment 1 and 2) of subject 8 were used against the same set of controls. The mean ± SD of two experiments each performed in triplicate is shown. Results of individual experiments are shown in Supplementary Fig. 4. Two-way ANOVA followed by Dunnett *post-hoc* test: ^**^P ≤ 0.01, ^***^P ≤ 0.001. bp base pair, cDNA complementary DNA, Ctrl. 4–6 leukocyte controls, Ex exon, H_2_O water control of the RT-PCR.

We next investigated the effect of the homozygous c.360+1G>T change in intron 1 on *TBC1D2B* pre-mRNA splicing by using leukocyte-derived RNA from subject 8. We used a primer pair with the forward primer located in exon 1 and the reverse primer in exon 3 in RT-PCR experiments (Supplementary Table 1). We generated an RT-PCR product in subject 8 that was about 35 bp smaller than the wild-type amplicons generated from control-derived cDNAs (Fig. 3A). Direct sequencing of the subject 8-derived amplicon revealed *TBC1D2B* mutant transcripts with loss of the last 33 bp of exon 1 (r.328_360del) due to activation of a cryptic splice donor site in exon 1 (Fig. 3B). *TBC1D2B* r.328_360del mRNAs could lead to potential production of a TBC1D2B mutant protein which lacks 11 amino acid residues from valine 110 to lysine 120 [p.(Val110_Lys120del)] (Fig. 3B). Quantitative real-time PCR identified a statistically significantly reduced *TBC1D2B* mRNA level to 15–21% in leukocytes of subject 8 compared with controls (Fig. 3C and Supplementary Fig. 4). Drastic decrease of *TBC1D2B* total mRNA level in leukocytes from subject 8 suggests expression of other aberrantly spliced *TBC1D2B* transcripts in addition to *TBC1D2B* r.328_360del mRNAs from both *TBC1D2B* c.360+1G>T alleles. These other aberrantly spliced *TBC1D2B* mRNAs may be efficiently degraded by NMD in subject 8-derived leukocytes, as they cannot be detected by qualitative and quantitative RT-PCR analyses. Potential production of a TBC1D2B p.(Val110_Lys120del) mutant protein could not be analyzed due to lack of patient-derived fibroblasts from subject 8.

### Clinical findings in ten subjects

In Table 1, we briefly summarized clinical and neuroimaging features of all ten subjects carrying biallelic *TBC1D2B* variants known to date. These include new follow-up data from subjects 1, 3, and 4 [1], previously published data from subjects 5 and 6 [2], and data from subjects 7 to 11 identified in this study. Clinical case reports of the five new patients and comprehensive clinical data of the ten subjects can be found in Supplementary information and Supplementary table 3. We here summarize commonly observed clinical features of subjects 1 and 3 to 11.

#### Abnormality of the face

**Fig. 4:**
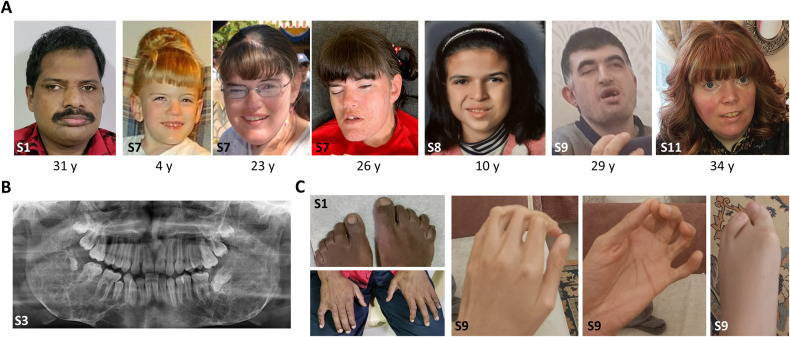
Clinical photographs of subjects with biallelic variants in *TBC1D2B*. **A** We assessed the facial features of subjects for whom photographs were available and for whom authorization has been obtained for disclosure. From left to right: facial photographs of subject 1 (S1) at age 31 years, subject 7 (S7) at 4 years (after gingival hyperplasia surgery), 23 years, and 26 years (a few days before she died), subject 8 (S8) at 10 years, subject 9 (S9), subject 8’s brother, at 29 years, and subject 11 (S11) at 34 years. All subjects except subject 11 had mandibular prognathia. Subject 1 had a coarse face with prominent nose, low hanging columella, and pigmented freckles on face. The face of subject 7 changed over time and the mandible became more prominent. At the age of 23 years, subject 7 had brown freckles on her face. Subject 8 had facial asymmetry and a low hanging columella. Low hanging columella was also present in subject 9. Subject 11 had mild hypertelorism, long palpebral fissures, mild eversion of the lateral lower eyelids, short nose with anteverted nares, smooth philtrum, and thin upper lip vermilion. She also had a mildly prominent premaxillary region. She presented with a malar rash and bilateral infraorbital xanthelasma. **B** Orthopantogram of subject 3 (S3) done at age 11 years shows extensive fibrous dysplasia of the mandible, which is stable when compared to previous orthopantogram done at age 8.5 years [compare with Fig. 1c published in Harms et al.] [1], (**C**) Finger and toe abnormalities in subjects 1 and 9. Photographs of hands and feet of subject 1 show flexion contractures of fingers and toes at the age of 31 years. Photographs of left hand and left foot of subject 9 at the age of 29 years show flexion contractures of fingers and tibial deviation of the 4th toe. S, subject; y, years.

Five subjects had coarse facial features (Fig. 4A). Eight subjects had gingival overgrowth that started early in childhood and required surgery in a few individuals. An abnormal mandible morphology was found in eight subjects. Five subjects had a prominent mandible (Fig. 4A), while three subjects had been diagnosed with fibrous dysplasia of the mandibular bones (Fig. 4B and [2]). In two subjects (brothers), soft tissue growth also involved the maxilla region [2]. Cherubism had been diagnosed in two subjects [1, 2]. Pigmented freckles appeared on the face of two subjects in adulthood.

#### Abnormality of the nervous system

Five subjects had a normal development in childhood, while developmental delay was present in the other five subjects. Mental deterioration or cognitive regression became apparent in six subjects between the ages of 5 and 20 years and was associated with gait ataxia and slurred speech. Two subjects had no speech development, and two other subjects were unable to speak in their twenties and thirties. Eight subjects developed seizures between the ages of 4 months and 32 years that were controlled with medication in four. Five subjects showed behavioral abnormalities. Brain abnormalities included cerebellar and/or cerebral atrophy in six subjects and lateral ventricle dilatation in three subjects. Movement abnormalities were reported in six subjects, including tremors, dystonia, and myoclonus. One subject could not walk as a toddler, and two adult subjects could walk with assistance. Three subjects became bedridden between the ages of 12 and 36 years and had respiratory failure requiring assisted ventilation.

#### Abnormality of the eye

Visual loss was noted in five subjects between the ages of 3 and 20 years. One subject had complex ophthalmologic abnormalities, in part due to pre-maturity. Macular optical coherence tomography revealed inner layer schisis-like cavitation as well as vacuoles and deposition in ganglion cell layer in one subject.

#### Additional abnormalities

Two subjects had bilateral hearing loss, and one had minor hearing issues. Seven subjects had flexion contractures involving fingers, toes, elbows, knees, arms, and/or legs (Fig. 4C), which began early in two, at 3–5 years of age. Five subjects had low blood cell counts, with two subjects with pancytopenia and one subject each diagnosed with thrombocytopenia, leukopenia or mild lymphopenia and thrombocytopenia. Seven subjects were alive; the oldest were 31 and 35 years old. Three subjects died between the ages of 21 and 39 years.

## Discussion

With the five additionally described subjects with biallelic *TBC1D2B* variants, 12 different disease-associated *TBC1D2B* variants have now been reported, including seven nonsense, three frameshift, one splice site, and one missense variant (Supplementary Table 2). The nonsense, frameshift, and splice site variants likely represent loss-of-function alleles and lead to TBC1D2B deficiency when present in a homozygous or compound heterozygous state in subject fibroblasts [1]. In fibroblasts of subject 7 with compound heterozygous *TBC1D2B* variants c.2758C>T; p.(Arg920*) and c.2584C>T; p.(Arg862Cys), normal *TBC1D2B* mRNA level as well as TBC1D2B protein level of about 50% compared with control cells were detected. Any C-terminally truncated TBC1D2B protein that may be produced is likely to be unstable and degraded. In contrast, the data indicate the TBC1D2B-Arg862Cys mutant protein is normally produced and stable, and may have an impaired GAP activity. In leukocytes of subject 8 with the homozygous c.360+1G>T variant, aberrantly spliced *TBC1D2B* mRNAs in addition to drastically reduced *TBC1D2B* mRNA level were detected. Together, the disease-associated *TBC1D2B* alleles identified up to date likely lead to complete loss of TBC1D2B or significantly impaired TBC1D2B function.

By analyzing clinical features of ten subjects with biallelic *TBC1D2B* loss-of-function variants we delineated a core phenotype. Cognitive and motor development of affected individuals can be normal or delayed, and most of the subjects develop seizures. Gingival overgrowth usually starts in early childhood. Mandibular prognathia is a common feature, with fibrous dysplasia of the mandible and/or maxilla in a few subjects. Progressive neurological deterioration was observed and begins between the ages of 10 and 20. Mental deterioration can be severe, and death can occur in early adulthood. Flexion contracture is a common feature. Visual loss and low blood cell counts have been observed in half of the subjects. Together, the *TBC1D2B* disorder is a progressive neurological disease with gingival overgrowth and mandibular prognathia or cherubism as characteristic associated abnormalities. Hematological abnormality has not yet been reported and seems to be a novel feature associated with TBC1D2B deficiency. The combination of manifestations makes *TBC1D2B* disorder a clinical recognizable phenotype, at least in subjects who have developed progressive disease with neurological deterioration. Although all *TBC1D2B* pathogenic variants reported to date likely are loss-of-function variants, the phenotypic presentation in the ten subjects is variable. For example, the 35-year-old subject 11 with the homozygous early stop variant p.(Glu9*) had a relatively mild phenotype, whereas subject 7 with a nonsense and a missense variant in trans presented with severe mental deterioration and decline and died at the age of 26 years and 10 months. In the literature, a 21-year-old man and an 11-year-old girl with clinical features overlapping those of the *TBC1D2B* disorder have been reported, including gingival overgrowth since early childhood, osteofibrosis of the maxillary alveolar bone or cherubism, mental or psychomotor retardation, and cerebral and/or cerebellar atrophy. Chromosome analysis revealed a normal karyotype in both subjects [12, 13]. Genetic analysis of the *TBC1D2B* gene would be valuable in the two subjects to potentially identify additional individuals with this rare disorder.

Fibrous dysplasia of the mandible and/or maxilla was observed in three subjects and diagnosed as cherubism in two (Supplementary Table 3). Cherubism is characterized by bilateral proliferative fibro-osseous lesions limited to the mandible and maxilla. Usually, this is an isolated childhood-onset, self-limited bone disease without other physical abnormalities [14]. Isolated cherubism is an autosomal dominant condition and caused by heterozygous missense variants in *SH3BP2*, mainly affecting codons 415 and 418–420 and causing a gain-of-function effect [15–18]. SH3BP2 is a cytoplasmic adapter protein and binds to a range of proteins, including CIN85 and HIP-55 that are involved in endocytic and cytoskeletal regulation [19]. We hypothesize that a possible function of SH3BP2 in intracellular trafficking may overlap with the proposed role of TBC1D2B in membrane fusion and vesicle trafficking [1, 8].

Clinical features of subjects with biallelic *TBC1D2B* pathogenic variants show phenotypic overlap with syndromic neurodevelopmental K^+^ channelopathies or Zimmermann-Laband syndrome associated with dominant *KCNN3*, *KCNH1*, and *KCNK4* variants. Affected individuals present with developmental delay and/or intellectual disability, coarse facial features, gingival overgrowth, hypoplastic nails and/or distal phalanges, and hypertrichosis [20]. However, subjects with biallelic *TBC1D2B* variants did not have hypertrichosis and hypoplastic distal phalanges of fingers and/or toes. Ramon syndrome, another rare condition, characterized by intellectual disability, seizures, cherubism, gingival overgrowth, hypertrichosis, and short stature [21–28], also shows clinical overlap with the subjects reported here. While a hallmark of the *TBC1D2B* disorder is mental deterioration associated with cerebellar and/or cerebral atrophy and lateral ventricular dilatation, developmental or cognitive regression has not been described in Ramon syndrome [22, 26, 28].

With the recent data that TBC1D2B positively regulates autophagy [29], an imbalanced crosstalk between autophagy and the endolysosomal pathway can be assumed in subjects with biallelic *TBC1D2B* variants [30]. Importantly, defects in autophagy and the endolysosomal system have been associated with neuronal dysfunction and neurodegenerative diseases [31, 32], raising the possibility that the *TBC1D2B*-related disease belongs to this group of disorders.

### Supplementary information

Supplemental Material

## Data Availability

The data that supports the findings of this study are available within the paper and in the supplementary information. Genome and exome sequencing data are not publicly available due to privacy or ethical restrictions. The novel *TBC1D2B* variants reported in subjects 7–11 in this manuscript were submitted to the LOVD database (https://databases.lovd.nl/shared/genes/TBC1D2B), with the LOVD variant IDs: #0000927486, #0000927487, #0000927488, #0000927489, #0000952085, #0000952086, and #0000952087.
