# Supplementary material for: Loss of TBC1D2B causes a progressive neurological disorder with gingival overgrowth
Source: Eur J Hum Genet. 2024 Feb 19;32(5):558–66. doi: 10.1038/s41431-024-01563-5 (PMC11061173; doi:10.1038/s41431-024-01563-5)
Supplement: Supplementary file 1 — Supplemental Material [file 41431_2024_1563_MOESM1_ESM.docx]

**Supplementary Information**

**Loss of TBC1D2B causes a progressive neurological disorder with gingival overgrowth**

Frederike L. Harms,^1^ Jessica Erin Rexach,^2^ Stephanie Efthymiou,^3^ Busra Aynekin,^3^ Hüseyin Per,^4^ Ayten Güleç,^4^ Sheela Nampoothiri,^5^ Hugo Sampaio,^6,7^ Rani Sachdev,^8,9^ Radka Stoeva,^10^ Kasiani Myers,^11,12^ Loren D. M. Pena,^12,13^ Theodosia A. Kalfa,^12,14^ Marisa Chard,^15,16^ Megan Klassen,^15^ Megan Pries,^15^ Kerstin Kutsche^1^

1 Institute of Human Genetics, University Medical Center Hamburg‐Eppendorf, Hamburg, Germany

2 Department of Neurology, Program in Neurogenetics, David Geffen School of Medicine, University of California Los Angeles, Los Angeles, California, USA

3 UCL Queen Square Institute of Neurology, London, WC1N 3BG, United Kingdom

4 Division of Pediatric Neurology, Department of Pediatrics, Faculty of Medicine, Erciyes University, Kayseri, Turkey

5 Department of Pediatric Genetics, Amrita Institute of Medical Sciences and Research Centre, Cochin, Kerala, India

6 Department of Women and Children's Health, University of New South Wales, Randwick Campus, Randwick, NSW, Australia

7 Sydney Children's Hospital, Randwick, NSW, Australia

8 Centre for Clinical Genetics, Sydney Children’s Hospital, Randwick, NSW, Australia

9 School of Women’s and Children’s Health, University of New South Wales, Randwick, NSW, Australia

10 Department of Medical Genetics, Le Mans Hospital, Le Mans, France

11 Division of Bone Marrow Transplant, Cincinnati Children’s Hospital Medical Center, Cincinnati, Ohio, USA

12 Department of Pediatrics, University of Cincinnati College of Medicine, Cincinnati, Ohio, USA

13 Division of Human Genetics, Cincinnati Children’s Hospital Medical Center, Cincinnati, Ohio, USA

14 Division of Hematology, Cincinnati Children’s Hospital Medical Center, Cincinnati, Ohio, USA

15 Provincial Medical Genetics Program, Newfoundland and Labrador Health Services, St. John’s, NL, Canada

16 Department of Pediatrics, Memorial University Faculty of Medicine, St. John’s, NL, Canada

Correspondence to:

Kerstin Kutsche, PhD

Institute of Human Genetics

University Medical Center Hamburg-Eppendorf

Martinistraße 52

20246 Hamburg

Germany

email: kkutsche@uke.de

**Clinical case reports**

Subject 7 was born premature at age 28 weeks complicated by retinopathy of prematurity. She presented with global neurodevelopmental delay particularly in the motor and language domains. She had refractory epilepsy consistent with Lennox-Gastaut syndrome with seizures beginning at 9 months of age. She subsequently developed prominent recurrent gingival overgrowth beginning at 18 months of age. She had progressive flexion contractures of the toes requiring surgical release at 10 years of age. She also presented with visual problems, including retinal detachment, proliferative vitreoretinopathy, and phthisis bulbi with metastatic bone formation requiring enucleation at 15 years of age. Despite these delays and symptoms, she was described as very active, playful, and engaged. At 14 years of age, her seizures stopped, seemingly spontaneously, and she developed obsessive compulsive disorder with impaired social awareness and loss of empathy. At 20 years of age, she started to regress and developed new traits that could not be explained at the time. She regressed in language from speaking 2-3 word sentences with a vocabulary of 100 words to having minimal speech output. She lost her motor skills and became increasingly weak, spastic, and ataxic such that she became wheelchair bound. Extensive workup was performed and negative for an acquired cause of the regression, most notably negative EEGs, autoimmune disease marker panels, EMG/NCS, infectious and metabolic workup, and chromosomal microarray. Her brain MRI at age 18 years was read as possible cerebral cortical atrophy, but subsequent scans appeared stable up to age 25 years. Concurrent with these changes, she developed additional systemic traits without any identifiable cause, including prominent freckling on her face, frequent maculopapular rashes on her body, thrombocytopenia, low protein, low albumin, low immunoglobulin (IgG), low calcium, and elevated C-reactive protein. Her mandibular prominence became increasingly more pronounced. She continued to progressively decline. She presented for genetic testing. She passed away of complications of her motor decline at age 26 years and 10 months.

Subjects 8 and 9 were born to consanguineous parents (cousins). Subject 8 was a girl who played volleyball in her school team and was generally healthy until the age of 8 years except for a history of epileptic seizures at 2 years of age. At the age of 8 years, she experienced dystonic attacks and was diagnosed with diabetes mellitus two years later. Subsequently, she developed speech impairment and cognitive decline, and cerebral atrophy was discovered on brain imaging. Her walking ability deteriorated, leading her to become bedridden at the age of 12 years. She underwent surgery for gingival overgrowth with subsequent changes noted in her facial and mandibular morphology. She developed flexion contractures (fingers and toes affected) at about the age of 12 years and required tracheostomy and mechanical ventilation support in the same year. She had a left focal clinical seizure and corresponding right hemispheric epileptic discharges on EEG. Since then, she had been taking antiepileptic drugs. She did not seek hospital treatment after the age of 18 years due to social reasons, but her family reported that she lost her vision at the age of 20 years. She died of aspiration pneumonia and sepsis at the age of 21 years.

Subject 9, the brother of subject 8, was born vaginally at 39 - 40 weeks of gestation and was hospitalized for meconium aspiration/asphyxia. He had microcephaly at birth and developmental delay from infancy. He could never speak. He had a tip-toe gait and was operated for this at the age of 2 years. He could walk with help at about 4-5 years and without help after the age of 14 years. He always had a low white blood cell count. He had tibial deviation of the 4th right toe since early childhood and flexion contractures of fingers and toes after the age of 4-5 years. When he was about 20 years old, he could not see. He underwent cataract surgery and three months after the surgery, he lost his vision again and needed assistance to walk.

Subject 10 is a 10-year-old European-American girl who was born to non-consanguineous parents after a 37-week gestation complicated by pre-eclampsia. She appeared healthy up to late infancy when she was noted to have developmental delays in speech and motor areas. She presented with generalized tonic-clonic seizures at 13 months of age and was diagnosed with Lennox-Gastaut syndrome, challenging to control. She was started on topiramate. Parents noted additional involuntary jerky movements (myoclonic jerks), which improved with the addition of levetiracetam. Brain MRI at 18 months of age showed small craniofacial ratio and non-specific white matter signal changes compatible with microcephaly and either delayed myelination, dysmyelination, or non-specific leukodystrophy. She developed gingival hyperplasia, unlikely to be associated with her anti-convulsant treatment. Speech development was significantly delayed such that her vocabulary was limited to four words at 3 years of age. With speech therapy and excellent parental care, she continued to make slow gains. At 10 years of age, she knew the alphabet but did not read. She could count to 20 and communicate with about 30 words and some sign language. She could follow one step instructions consistently, and simple two-step instructions with more difficulty. At 8 years of age, pancytopenia was identified on screening laboratory studies. She progressed to transfusion-dependence for both platelets and red cells, and she also had severe neutropenia. Bone marrow examination at that time showed normal cellularity (80% overall) with cellular atypia in the erythroid lineage without evidence of myelodysplasia, consistent with ineffective erythropoiesis. Bone marrow also showed a high proliferative index demonstrated by tetraploidy in 4 out of 20 cells on cytogenetics and 6-8% by fluorescence *in situ* hybridization (FISH). She was started on scheduled red cell transfusions to suppress ineffective hematopoiesis as well as eltrombopag and concurrent iron chelation. It was expected she would need a bone marrow transplant, however, her blood counts instead gradually improved. After one year, she achieved transfusion independence and resolution of neutropenia with normalization of bone marrow studies and improvement of tetraploidy to 1% by FISH. Her thrombocytopenia improved to moderate levels (~60 x 103 platelets/µL), but did not fully resolved. She was also found to have low IgG levels for which she was treated with intravenous IgG supplementation every month.

Subject 11 was born at term. Her parents were not known to be consanguineous. She was diagnosed with infantile spasms at 4 months of age. Her seizures were refractory to medications in early childhood but were well controlled by her teen years. She met all early developmental milestones on time, however she was noted to be cognitively behind her peers on Kindergarten entry at 5 years of age. This was followed by a gradual decline in cognitive functioning that required placement into a “challenging needs” classroom by age 10 years. She started having episodes of psychosis at age 25 years and had what was described as an episode of cataplexy. She was treated with electroshock therapy and antipsychotic medications and the psychosis resolved. Most of her antiepileptic medications were discontinued at that time, as she continued to be seizure free for years. However, she subsequently had a recurrence of multiple seizures at age 34 years. While hospitalized for recurrent seizures, she was witnessed on cardiac monitor to have brief period of asystole followed by third degree heart block during a prolonged seizure episode. She was known to have a prior history of left bundle branch block. Echocardiogram was normal. Based on this episode, a pacemaker was placed. She was assessed by Medical Genetics for the first time at age 34 years. At age 35 years, she ambulated independently and could speak clear sentences. Due to cognitive impairment, she required constant supervision of her family. She completed most activities of daily living with some assistance. Neurological exam was also notable for intermittent nystagmus and intermittent hand temor. She did not demonstrate clinical symptoms of impaired vision, and has not had a formal ophthalmological exam. Her blood counts showed intermittent low platelets and chronic mild lymphopenia. Her clinical course was notable for some improvement in vocabulary and memory in recent years being reported by the family.

**Methods**

**Exome and genome sequencing**

**Subject 7**

Exome sequencing (ES) on DNA samples of subject 7 and parents was performed using the Agilent SureSelect Clinical Research Exome XT kit (Agilent) and an HiSeq 2500 platform (Illumina). In total 8,240,590,752 bases of sequence were generated and uniquely aligned to the human reference genome hs37d5 (1000genomes Phase2 Reference Genome Sequence based on NCBI GRCh37 that includes the Revised Cambridge Reference Sequence [rCRS] NC_012920 mitochondrial genome sequence), generating a mean coverage of 91x per base within the RefSeq protein coding bases of the human genome. Approximately 99% of the mitochondrial genome was covered to a depth of >=4x. In total 67,295 DNA variants were identified, including 60,764 single nucleotide substitutions and 6,531 small deletions/insertions (1-10bp). Only clinically significant variants which are associated with the primary clinical concern(s) were reported.

**Subject 8**

Genomic DNA was extracted from peripheral blood samples of subjects 8 and 9 and parents according to standard procedures of phenol-chloroform extraction. Single ES on DNA of subject 8 was performed as described elsewhere [1] in Macrogen, Korea. Briefly, target enrichment was performed with 2 μg genomic DNA using the SureSelectXT Human All Exon Kit version 6 (Agilent) to generate barcoded whole-exome sequencing libraries. Libraries were sequenced on the HiSeqX platform (Illumina) with 50x coverage. Quality assessment of the sequence reads was performed by generating QC statistics with FastQC. The bioinformatics filtering strategy included screening for only exonic and donor/acceptor splicing variants. In accordance with the pedigree and phenotype, priority was given to rare variants (<0.01% in public databases, including 1000 Genomes project, NHLBI Exome Variant Server, Complete Genomics 69, and Exome Aggregation Consortium [ExAC v0.2]) that were fitting a recessive (homozygous or compound heterozygous) or a *de novo* model and/or variants in genes previously linked to spasticity, developmental delay, intellectual disability, and other neurological disorders.

**Subject 10**

Genomic DNA was extracted from peripheral blood samples of subject 10 and parents according to standard procedures of phenol-chloroform extraction. Genome sequencing (GS) trio on DNA of subject 10 and her parents was performed at the Broad Clinical Labs (Cambridge, MA, USA). Briefly, after PCR-free sample preparation, sequencing was performed on the Illumina NovaSeq X Plus instrument to produce research PCR-Free GS with 30x short read coverage. The reads were mapped against GRCh38 human reference using BWA software. The joint genotyping variant calls were produced using GATK Haplotype caller software on Terra.bio cloud platform. The variant analysis was performed via QIAGEN Clinical Insight (QCI®) Interpret Translational cloud-based software. Rare variants defined as <0.01% in public databases, including gnomAD, 1000 Genomes project, NHLBI Exome Variant Server, and Exome Aggregation Consortium (ExAC v0.2) that were fitting a recessive (homozygous or compound heterozygous) or a *de novo* model of inheritance were prioritized. Further gene to disease prioritization was given to genes previously linked to global developmental delay, mild intellectual disability, intractable seizures, pancytopenia, delayed speech and language development. The candidate variant calls were visually checked using IGV [2].

**Subject 11**

Genomic DNA was extracted from peripheral blood samples of subject 11 and parents. ES-trio on DNA of subject 11 and her parents was performed at Blueprint Genetics (Espoo, Finland). The reads were mapped to the human reference genome (GRCh37/hg19). Burrows-Wheeler Aligner (BWA-MEM) software was used for read alignment. Variant data was annotated using VcfAnno and VEP with a variety of public variant databases including but not limited to gnomAD, ClinVar and HGMD. The clinical evaluation team assessed the pathogenicity of identified variants by evaluating the information in the patient referral, reviewing the relevant literature, and manually inspecting the sequencing data if needed. All coding region *de novo* variants were evaluated, novel heterozygous, truncating variants (nonsense, frameshift, canonical splice site variants) in genes predicted to be intolerant for loss-of-function variation based on gnomAD variant data, rare truncating homozygous or (predicted) heterozygous variants, or a combination of rare truncating and rare missense variant that is predicted deleterious by multiple *in silico* tools. In addition, only variants in genes whose known expression pattern and function are considered relevant for the phenotype are included.

**Variant segregation and validation**

Confirmation of the *TBC1D2B* variants in fibroblast-derived DNA of subject 7 (**Fig. 1A**) and segregation of the *TBC1D2B* c.360+1G>T variant in members of family 6 (subjects 8 and 9 and their parents; **Supplementary Fig. 1**) were performed by Sanger-sequencing. Primers designed to amplify the selected regions of *TBC1D2B* (NM_144572.2) are described in **Supplementary Table 1**. Amplicons were directly sequenced using the ABI BigDye Terminator Sequencing Kit (Applied Biosystems) and an automated capillary sequencer (ABI 3500, Applied Biosystems). Sequence electropherograms were analyzed using the Sequence Pilot software (JSI Medical Systems).

**Cell culture of primary dermal fibroblasts**

Primary dermal fibroblasts were obtained from a skin biopsy of subject 7 and three female control individuals. Fibroblasts were cultured in Dulbecco’s modified Eagle medium (DMEM; Thermo Fisher Scientific) supplemented with 10% fetal bovine serum (FBS; GE Healthcare) and penicillin-streptomycin (100 U/mL and 100 mg/mL, respectively; Thermo Fisher Scientific). For all experiments, the same passage number of subject and control fibroblasts was used. Primary fibroblasts were regularly tested for mycoplasma contamination and confirmed to be mycoplasma free.

**RNA isolation, cDNA synthesis, RT-PCR and Sanger-sequencing, and quantitative real-time PCR (RT-qPCR)**

Total RNA was extracted from cultured primary fibroblasts of subject 7 and controls (Monarch Total RNA Miniprep kit, New England BioLabs). Leukocyte-derived RNA from PAXgene blood RNA tubes of subject 8 and three healthy controls was isolated using the PAXgene Blood RNA Kit IVD (Qiagen). RNA concentration and purity of the samples were assessed by use of the Microplate Spectrophotometer Epoch (BioTek). 1 µg total RNA was reverse transcribed (LunaScript RT Super Mix kit, New England BioLabs). Primer sequences for *TBC1D2B* transcript analysis are in **Supplementary Table 1**. RT-PCR products were directly Sanger-sequenced. Technical triplicates of RT-qPCR samples were prepared as a 10 µL approach with the SYBR Green I-based Luna Universal qPCR Master Mix (New England BioLabs), 500 nM of each primer, and 1 µl of the reverse transcription reaction. Primer sequences for RT-qPCR are described in **Supplementary** **Table 1**. RT-qPCR was performed using the QuantStudio 3 Real-Time PCR System (Thermo Fisher Scientific) equipped with QuantStudio Design&Analysis Software v1.4.3 (Thermo Fisher Scientific). The PCR conditions included a pre-run at 95°C for 5 min, followed by 40 cycles of 30 s at 95°C, 30 s at 58°C and 45 s at 72°C. PCR amplification specificity was determined by melting curve analysis with a range from 60°C to 95°C. The values of the cycle threshold (CT) of the target mRNAs were normalized to the mRNA of *GAPDH*. For relative gene expression, the comparative cycle threshold (ΔΔCT) values were calculated with the QuantStudio Design&Analysis Software (Thermo Fisher Scientific) with *GAPDH* as housekeeping gene and expressed as x-fold change to control 1 or control 4.

**Immunoblotting**

Fibroblasts of subject 7 and controls were harvested in ice-cold RIPA buffer (50 mM Tris-HCl, pH 8.0; 150 mM NaCl; 1% NP-40; 0.5% DOC [sodium deoxycholate]; 0.1% SDS [sodium dodecyl sulfate]) supplemented with Mini Protease Inhibitor (Roche) and lysed for 10 min on ice. Cell debris was removed by centrifugation for 10 min. Protein extracts were separated on SDS-PAGE under denaturing conditions and transferred to polyvinylidene fluoride membranes. Membranes were blocked followed by incubation with a polyclonal rabbit anti-TBC1D2B primary antibody (#HPA052663; antibody recognition region: amino acid residues 372-452; Sigma-Aldrich; 1:1,000) overnight at 4°C. After washing, membranes were incubated with StarBright Blue 700 dye-linked anti-rabbit secondary antibody (#12004161; BioRad; 1:10,000) together with hFAB Rhodamine anti-tubulin (#12004165; Bio-Rad; 1:10,000) at room temperature for 1 h. After washing, immunoblots were digitally imaged using a ChemiDoc MP (Bio-Rad). Exposure time was optimized to avoid saturation. Bands were automatically defined and intensities were determined using the build-in band detection tool of the Image Lab v6.0 software (Bio-Rad).

**Subject 8**

**DNA**

**DNA**

**Leukocytes**

**c.360+1G>T**

**Subject 9**

**Mother**

**Father**

**DNA**

**DNA**

**Supplementary Figure 1. *TBC1D2B* variant validation in subject 8 and segregation in members of family 6.** Validation and segregation of the *TBC1D2B* c.360+1G>T variant in leukocyte-derived DNA of subjects 8 and 9 and their parents (family 6). Partial sequence electropherograms demonstrating the *TBC1D2B* c.360+1G>T variant in leukocyte-derived DNA of subjects 8 and 9 in the homozygous state. Their parents (mother and father) are heterozygous carriers of the *TBC1D2B* variant. Arrows point to the position of the pathogenic variant.

**
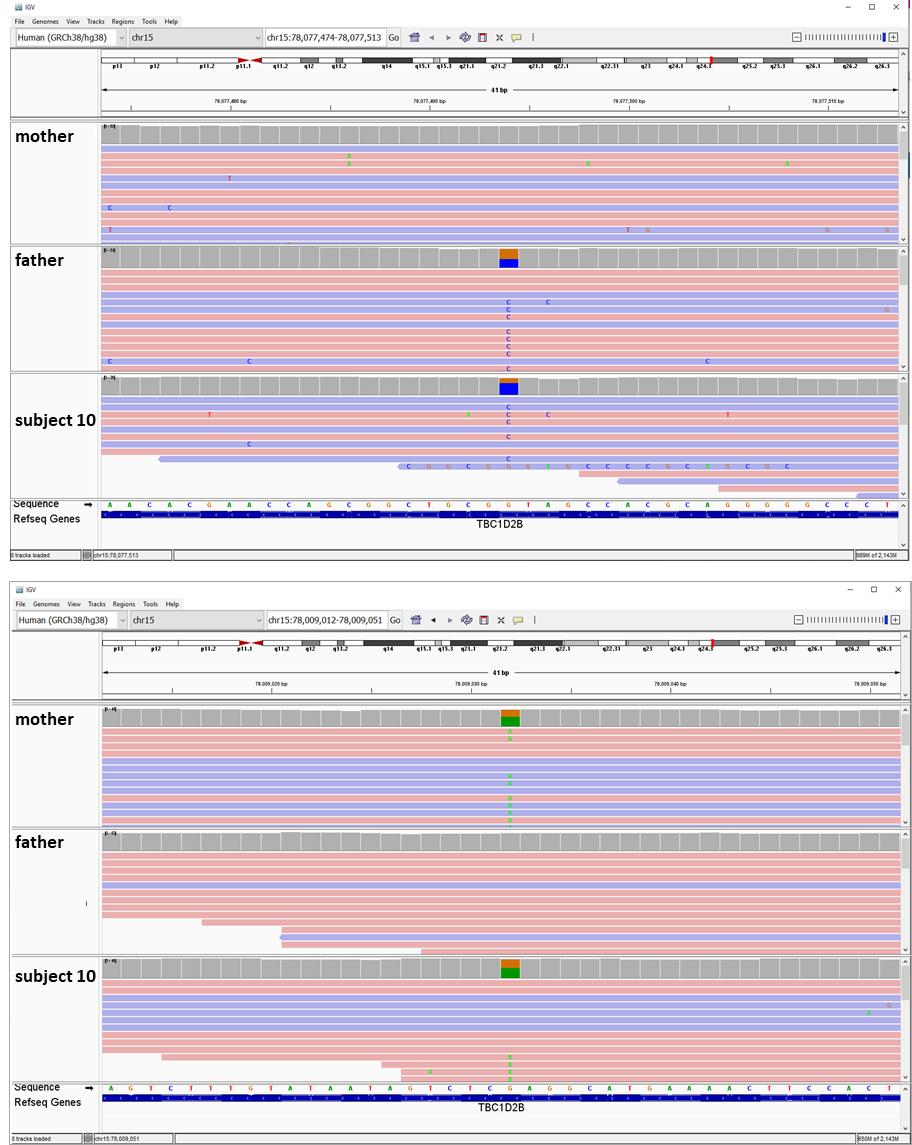
**

**Supplementary Figure 2. Visualization of the *TBC1D2B* variants identified by trio genome sequencing using the Integrative Genomics Viewer (IGV) in subject 10 and her parents.** The proband was compound heterozygous for the paternally inherited *TBC1D2B* variant c.2353C>T; p.(Arg785*) (NC_000015.10:g.78009032G>A) and the maternally inherited *TBC1D2B* variant c.159C>G; p.(Tyr53*) (NC_000015.10:g.78077494G>C).


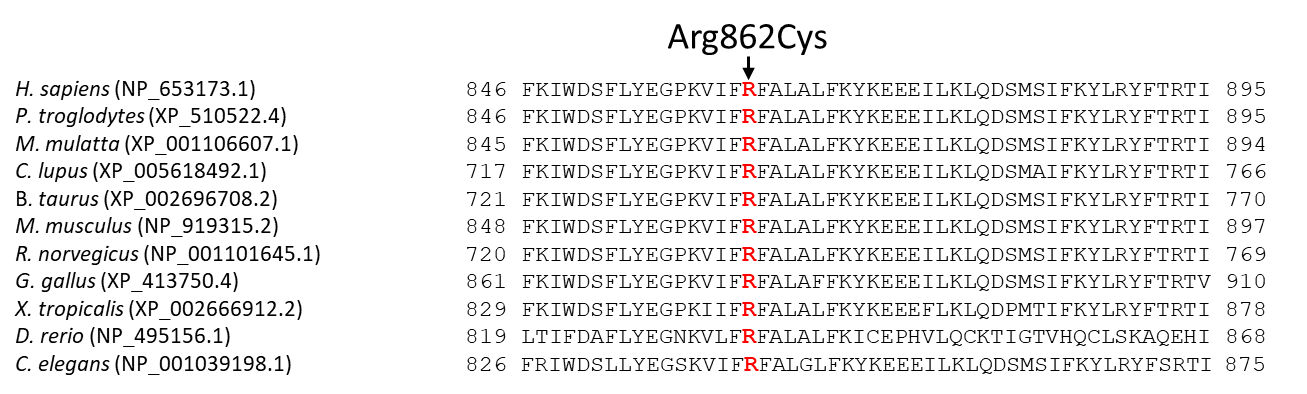


**Supplementary Figure 3. Partial amino acid sequence alignment of human TBC1D2B with orthologs in the single letter code.** Arginine 862, located in the RABGAP domain (amino acids 659-879) of TBC1D2B, shows high evolutionary conservation between species. Multiple alignment was gathered from http://www.ncbi.nlm.nih.gov/homologene/56694. Residue affected in subject 7 (Arg862Cys) is highlighted in red. RABGAP, RAB GTPase-activating protein (GAP) domain.

**
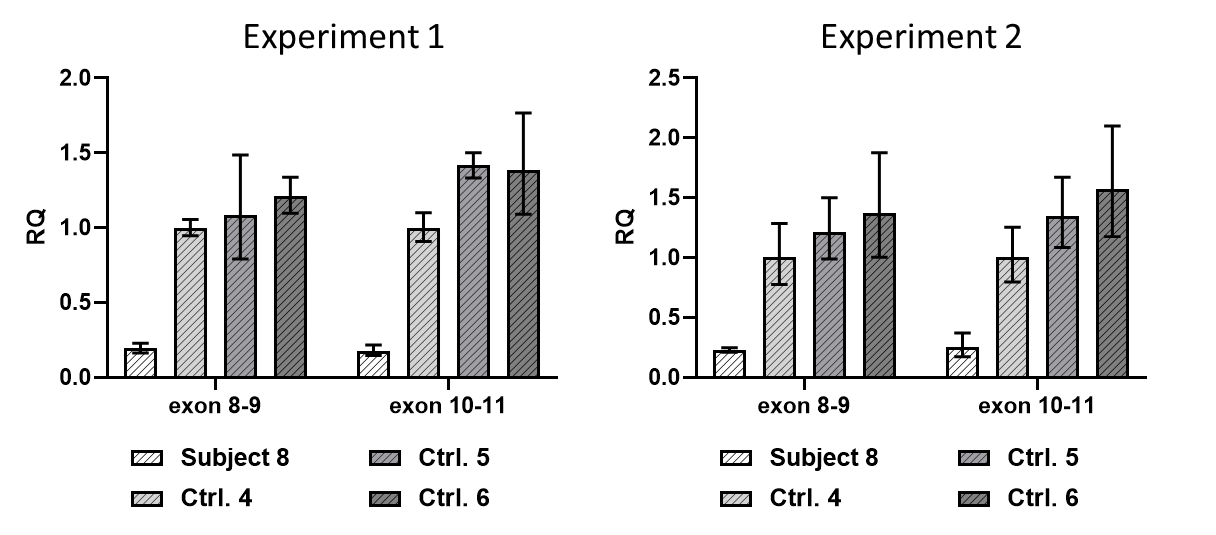
**

**Supplementary Figure 4. Individual data sets of two RT-qPCR experiments used for quantification in Fig. 2C.** Relative quantification of *TBC1D2B* mRNA levels determined by real-time quantitative PCR using two primer pairs (exons 8-9 and exons 10-11) with leukocyte-derived cDNA of subject 8 and three controls. Two independent RNA isolations (Experiment 1 and 2) from leukocytes of subject 8 were used against the same set of controls. Relative quantification (RQ) was performed according to the ΔC_T_ method. The amount of *TBC1D2B* mRNA relative to *GAPDH* mRNA is presented. The mean ± SD of technical triplicates is shown. Ctrl. 4-6, controls.

**Supplementary Table 1. Sequence of oligonucleotides used in this work**

| ***TBC1D2B* primer sequences for variant validation and segregation** | | | |
| --- | --- | --- | --- |
| **Template** | **Exon** | **Direction** | **Sequence (5’ → 3’)** |
| DNA | exon 1-intron 1 | forward | AGC CGC TGG TTC GTG TTC |
|  |  | reverse | TTG GAG GAA GGA GGG TGG AT |
|  | 12 | forward | AAC CAG CAG GGT TTA TTT TCC |
|  |  | reverse | CCC TCT CCA ACA CTG TAC ACC |
|  | 13 | forward | CTG TGT GTG AGC ACT CTC TGG |
|  |  | reverse | AGT GCA CTT TCA CCC TTT GG |
| ***TBC1D2B* primer sequences for transcript analysis** | | | |
| **Template** | **Exon** | **Direction** | **Sequence (5’ → 3’)** |
| cDNA | 1 | forward | GAC GCC TGC TTC AGC TAC C |
|  | 3 | reverse | CAG AAG CAT TTG GGT GTG G |
|  | 11 | forward | TGG ATA GTG TCG TTA GTG ACA TCC |
|  | 13 | reverse | AGT GCA CTT TCA CCC TTT GG |
| **Primer sequences for RT-qPCR on cDNA** | | | |
| **Gene** | **Exon** | **Direction** | **Sequence (5’ → 3’)** |
| *TBC1D2B* | 8 | forward | TGC TCC AAG AAA TGA AGA CAC CAG |
|  | 9 | reverse | CTT CCT CAT CAT CCT CAG GTA CAG |
|  | 10 | forward | GGT GTC TCG TTA CCA TAG TGG AAG |
|  | 11 | reverse | TCC ACA AAT ACC ACC AGA AAC CAG |
| *GAPDH* | 3 | forward | TGA CCC CTT CAT TGA CCT CAA C |
|  | 5 | reverse | GCA TCG CCC CAC TTG ATT TTG |

**Supplementary Table 2. Previously published and novel *TBC1D2B* pathogenic variants**

| Chr. | Genomic position (hg38) | Nucleotide change  in coding DNA  (NM_144572.2) | Variant at RNA level | Predicted effect on protein level  (NP_653173.1) | gnomAD v4.0.0:  MAF (%) | CADD | REVEL | M-CAP | MetaSNP | NMD prediction |
| --- | --- | --- | --- | --- | --- | --- | --- | --- | --- | --- |
| 15 | 78077628 | c.25G>T | r.? | p.(Glu9*) | 0.001198 | 34 | ─ | ─ | ─ | ─ |
| 15 | 78077494 | c.159C>G | r.? | p.(Tyr53*) | 0.000522 | 37 | ─ | ─ | ─ | ─ |
| 15 | 78077292 | c.360+1G>T | r.328_360del | p.(Val110_Lys120del) | absent | 34 | ─ | ─ | ─ | ─ |
| 15 | 78054122 | c.426dup | r.? | p.(Asn143*) | absent | 26.3 | ─ | ─ | ─ | This variant is subject to degradation by NMD |
| 15 | 78044989 | c.595del | r.? | p.(Val199Trpfs*22) | absent | 25.3 | ─ | ─ | ─ | This variant is subject to degradation by NMD |
| 15 | 78044925_  78044926 | c.658_659del | r.658_659del | p.(Leu220Glufs*6) | absent | 23.5 | ─ | ─ | ─ | This variant is subject to degradation by NMD |
| 15 | 78017948 | c.1480C>T | r.? | p.(Gln494*) | 0.000140 | 41 | ─ | ─ | ─ | ─ |
| 15 | 78009090 | c.2295C>G | r.2295c>g | p.(Tyr765*) | absent | 38 | ─ | ─ | ─ | ─ |
| 15 | 78009032 | c.2353C>T | r.? | p.(Arg785*) | 0.000138 | 45 | ─ | ─ | ─ | ─ |
| 15 | 78009007 | c.2378T>A | r.2378u>a | p.(Leu793*) | absent | 40 | ─ | ─ | ─ | ─ |
| 15 | 78001731 | c.2584C>T | r.2584c>u | p.(Arg862Cys) | absent | 33 | 0.706 | 0.084 | 0.765 | ─ |
| 15 | 77998294 | c.2758C>T | r.2758c>u | p.(Arg920*) | 0.000518 | 42 | ─ | ─ | ─ | ─ |

The functional impact of *TBC1D2B* variants was predicted by the Combined Annotation Dependent Depletion (CADD) tool, the Rare Exome Variant Ensemble Learner (REVEL) scoring system, the Mendelian Clinically Applicable Pathogenicity (M-CAP) Score, and Meta-SNP. CADD is a framework that integrates multiple annotations in one metric by contrasting variants that survived natural selection with simulated mutations. Reported CADD scores are phred-like rank scores based on the rank of that variant’s score among all possible single nucleotide variants of hg19, with 10 corresponding to the top 10%, 20 at the top 1%, and 30 at the top 0.1%. The larger the score the more likely the variant has deleterious effects; the score range observed here is strongly supportive of pathogenicity, with all observed variants ranking above ~99% of all variants in a typical genome and scoring similarly to variants reported in ClinVar as pathogenic (~85% of which score >15) [3]. REVEL is an ensemble method predicting the pathogenicity of missense variants with a strength for distinguishing pathogenic from rare neutral variants with a score ranging from 0-1. The higher the score the more likely the variant is pathogenic [4]. M-CAP is a classifier for rare missense variants in the human genome, which combines previous pathogenicity scores (including SIFT, Polyphen-2, and CADD), amino acid conservation features and computed scores trained on mutations linked to Mendelian diseases. The recommended pathogenicity threshold is >0.025 [5]. Meta-SNP is a meta-predictor of disease causing variants, which integrates four existing methods (PANTHER, PhD-SNP, SIFT, and SNAP). The score ranges from 0-1, with a pathogenicity threshold of >0.5 [6]. The NMDEscPredictor (https://nmdprediction.shinyapps.io/nmdescpredictor/) utilizing the 50 bp rule was used to predict nonsense-mediated mRNA decay of *TBC1D2B* frameshift variants [7]. Chr., chromosome; MAF, minor allele frequency; NMD, nonsense-mediated mRNA decay; ─, not available.

**Supplementary Table 3.** Detailed clinical characteristics of previously published and new subjects with biallelic *TBC1D2B* variants

|  | **Harms *et al.* (2020) [8]: published and follow-up data** | | | | | | |
| --- | --- | --- | --- | --- | --- | --- | --- |
|  | **Family 1** | | | **Family 2** | | **Family 3** | |
|  | **Subject 1** | | **Subject 2** | **Subject 3** | | **Subject 4** | |
| **General information** | | | | | | | |
| **Ethnicity** | Indian | | | European, Portuguese | | European, Chechen | |
| **Sex** | male | | female | female | | male | |
| **Age at last examination** | 27 y | Follow-up at: 31 y | 20 y | 8.5 y | Follow-up at: 12.5 y | 8 m | Follow-up at: 3.5 y |
| **Alive/Dead** | alive | alive | died at 25 y | alive | alive | alive | alive |
| ***TBC1D2B* variant**  **(NM_144572.2/** **NP_653173.1)** | homozygous  c.2378T>A ; p.(Leu793*) | | no DNA available for testing | compound heterozygous  c.426dup ; p.(Asn143*)  c.1480C>T; p.(Gln494*) | | compound heterozygous  c.658_659del; p.(Leu220Glufs*6)  c.2295C>G; p.(Tyr765*) | |
| **Consanguinity** | ─ | | ─ | ─ | | ─ | |
| **Abnormality of the face (HP:0000271)** | | | | | | | |
| **Coarse facial features (HP:0000280)** | + | + | + | ─ | ─ | ─ | no data |
| **Gingival overgrowth (HP:0000212)** | + progressive  age at onset: 5 y; not exposed to any medications inducing gingival overgrowth | +  gingival overgrowth stable | +  age at onset: 5 y; not exposed to any medications inducing gingival overgrowth | +  age at onset: 3 y; not exposed to any medications inducing gingival overgrowth | +  gingival overgrowth stable; secondary teeth fully erupted | ─ | +  age of onset: 3 y;  exposed to valproic acid and vigabatrin since age of 12 months |
| **Abnormal mandible morphology (HP:0000277)** | +  mandible prominent over chin area | +  mandibular prognathia | +  mandible prominent over chin area | +  fibrous dysplasia of the mandible (cherubism) | +  fibrous dysplasia of the mandible (cherubism) | ─ | ─ |
| **Numerous pigmented freckles (HP:0007587)** | +  on face | +  on dorsum of hands and on face | +  on face | ─ | ─ | ─ | ─ |
| **Abnormality of the nervous system (HP:0000707)** | | | | | | | |
| **Global developmental delay (HP:0001263)** | ─ | ─ | ─ | +  mild | +  mild | axial hypotonia  (cannot hold his head up) | +  severe developmental delay |
| **Mental deterioration (HP:0001268)** | +  age at onset: 12 y (deterioration of scholastic performance) | + | +  age at onset: 13 y (deterioration of scholastic performance) | ─ | ─ | ─ | ─ |
| **Slurred speech (HP:0001350)** | +  age at onset: 5 y | +  severe | + | ─ | ─ | ─ | ─  absent speech |
| **Gait disturbance (HP:0001288)** | +  gait ataxia | +  cannot walk without support | +  gait ataxia at 13 y | ─ | ─ | ─ | +  inability to walk |
| **Abnormality of movement (HP:0100022)** | +  tremor of hands at 19 y | + | +  tremor of upper limbs | not reported | not reported | not reported | not reported |
| **Seizures (HP:0001250)** | +  episode of seizure at 19 y; on medication since then | ─  controlled on medication | +  age at onset: 19 y | +  first episode at 18 m, focal onset with visual loss and auditory features, epilepsy phenotype aligns with panayiotopoulos syndrome | ─  epilepsy controlled with levetiracetam and carbamazepine | +  first episode at 3 m,  status epilepticus | ─  no seizures since the last 18 m (controlled on medication) |
| **EEG abnormality (HP:0002353)** | ─  normal with 12 y | no recent EEG | +  generalized epileptiform discharges | +  frequent right occipital epileptiform discharges | ─  no recent need for EEG | +  right occipital intercritical focus with several electrical seizures | +  isolated generalized discharges more pronounced in the posterior region at 3 y |
| **Behavioral abnormality (HP:0000708)** | +  excess speech, inappropriate laughter | +  severe emotional lability (inappropriate laughter and cry) | +  inappropriate laughter and cry | +  some lack of maturity in peer interaction | ─  improved | ─ | ─ |
| **CT brain abnormalities** | agenesis of inferior vermis with cerebellar vermis hypoplasia (at 23 y) | no recent CT | agenesis of inferior vermis with cerebellar vermis hypoplasia, generalized cortical atrophy (at 20 y) | normal;  facial CT: multiloculated lucent expansile lesions present within both sides of the mandible, relative sparing of the mandibular condyles and midline portions of the mandible bodies around the symphysis menti. Diagnosis of cherubism was made | no recent CT | enlargement of the ventricular system without hydrocephalus, thin temporal horns associated with diffuse parenchymal atrophy most notable in the frontotemporal regions with sparing of the cerebellum | no recent CT |
| **Brain MRI abnormalities** | cerebellar atrophy, small midbrain, *ex vacuo* dilatation of ventricles, prominent cerebellar folia, white matter hyperintensity in centrum semiovale (at 14 y) | no recent MRI | not done | normal at 2 y | no recent MRI | frontotemporal cerebral atrophy, lateral ventricular dilatation (4 mo) | at 3 y: lateral ventricular dilatation remains stable, Blake's pouch cyst, regression of the subdural hematoma detected on brain imaging at 1 y |
| **Eye and hearing abnormalities (HP:0000478; HP:0000364)** | | | | | | | |
| **Visual loss (HP:0000572)** | +  at 15 y | +  can read big letters of newspaper; bilateral upgaze palsy | +  at 19 y | ─ | ─ | ─ | +  no reaction to visual stimuli at 3 y |
| **Abnormal fundus morphology (HP:0001098)** | +  bilateral partial optic atrophy (at 14 y) | +  marked optic disc pallor bilaterally; retina and vessels clinically normal | +  bilateral primary optic atrophy, salt pepper appearance with peripheral pigment clumps; retinal pigment dystrophy (at 20 y) | +  left optic disc pallor noted at 8 y | +  unchanged | not done | ─  normal fundus morphology at 3 y |
| **Abnormal retinal morphology on macular OCT (HP:0030612)** | +  inner layer schisis-like cavitation, vacuoles and deposition in ganglion cell layer of retina (at 14 y) | +  same changes as described in the previous OCT | +  inner layer schisis-like cavitation, vacuoles and deposition in ganglion cell layer of retina (at 20 y) | not done | ─  no evidence of inner layer schisis-like cavitation, vacuoles or deposition in ganglion cell layer of retina | not done | not done |
| **Hearing impairment (HP:0000365)** | +  bilateral moderate to severe sensorineural hearing loss | + | no data | ─ | ─  no evidence of sensorineural hearing loss at 10.5 y | not done | +  bilateral mild to moderate hearing loss |
| **Additional abnormalities** | | | | | | | |
| **Flexion contracture (HP:0001371)** | +  age at onset: 17 y;  affected fingers and toes | +  2nd-5th toes bilaterally; 4th-5th fingers bilaterally | +  age at onset: 15 y; affected fingers and toes | ─ | +  contracture of elbows and fingers - stable with intervention | ─ | +  bilateral knee contractures at 3.5 y |
| **Respiratory failure requiring assisted ventilation (HP:0004887)** | ─ | ─ | ─ | ─ | ─ | ─ | ─ |
| **Abnormality of blood and blood-forming tissues (HP:0001871)** | not reported | not reported | not reported | not reported | not reported | not reported | not reported |
| **Other features** | ─ | brisk deep tendon reflexes in both knees | ─ | ─ | ─ | macrocephaly (+3 SD) | occipitofrontal circumference in the normal range at 3.5 y |

**Supplementary Table 3 (followed).** Detailed clinical characteristics of previously published and new subjects with biallelic *TBC1D2B* variants

|  | **Correia-Costa *et al.* (2022) [9]** | | **This study** | | | | | **Total**^a^ |
| --- | --- | --- | --- | --- | --- | --- | --- | --- |
|  | **Family 4** | | **Family 5** | **Family 6** | | **Family 7** | **Family 8** |  |
|  | **Subject 5** | **Subject 6** | **Subject 7** | **Subject 8** | **Subject 9** | **Subject 10** | **Subject 11** |  |
| **General information** | | | | | | | | |
| **Ethnicity** | Latin, Brazilian | | European, Norwegian/Polish | Turkish | | European-American (of German ancestry) | European (English/Irish ancestry) |  |
| **Sex** | male | male | female | female | male | female | female | 5 males  5 females |
| **Age at last examination** | 36 y | 25 y | 25 y | 18 y | 29 y | 10 y | 35 y |  |
| **Alive/Dead** | died at 39 y due to COVID-19 | alive, bedridden and with tracheostomy | died at 26 y 10 m  (dysphagia, aspiration) | alive | alive | alive | alive | 7 alive  3 died |
| ***TBC1D2B* variant**  **(NM_144572.2/** **NP_653173.1)** | homozygous  c.595del ; p.(Val199Trpfs*22) | | compound heterozygous  c.2584C>T;  p.(Arg862Cys),  c.2758C>T;  p.(Arg920*) | homozygous  c.360+1G>T | | compound heterozygous  c.159C>G;  p.(Tyr53*),  c.2353C>T;  p.(Arg785*) | homozygous  c.25G>T; p.(Glu9*) |  |
| **Consanguinity** | + | | ─ | + | | ─ | ─ | Consanguinity in 2 families |
| **Abnormality of the face (HP:0000271)** | | | | | | | | |
| **Coarse facial features (HP:0000280)** | + | + | ─ | + | + | ─ | ─ | 5/9 |
| **Gingival overgrowth (HP:0000212)** | +  age at onset: 3 y | +  age at onset: 3 y | + ^b^  age at onset: 18 m following dilantin exposure for one month; continued after discontinuation of dilantin; operated for gingival overgrowth at 4 y and 6 y; gingival overgrowth impacted wisdom teeth that could not come through | +  age at onset: not exactly known; her jaw and face changed slowly after 12 y; operated for gingival overgrowth at 18 y | ─ | +  age at onset: not exactly known but present since early childhood | ─ | 8/10  possibly drug-induced in 1 subject |
| **Abnormal mandible morphology (HP:0000277)** | +  fibrous dysplasia of the mandible at 3 y | +  fibrous dysplasia of the mandible (cherubism) | +  mandibular prominence  in childhood (by age 4 y); more prominent overtime (age 23 y) | +  mandible prominent over the chin area | +  mandible prominent over the chin area | +  prominent malar surface and upper lip | ─ | 8/10 |
| **Numerous pigmented freckles (HP:0007587)** | ─ | ─ | +  whole face having brown freckles that appeared at ~22 y; developed random skin rashes since age of 25 y | ─ | ─ | ─ | ─ | 2/10 |
| + |  |  |  |  |  |  |  |  |
| **Global developmental delay (HP:0001263)** | ─ | ─ | +  delayed ambulation (at 20 m), later in childhood ran frequently, rarely sat and then regressed; language delay: had > 100 words maximally in childhood that then declined, spoke in 2−3 word sentences that then declined | ─ | +  developmental delay since infantile period; microcephaly at birth; can understand easy things | +  developmental delay since infancy | ─ | 5/10 |
| **Mental deterioration (HP:0001268)** | +  age at onset: 15 y | +  age at onset: 15 y | +  age at onset: 20 y | +  speech problems started at age 10 y; bedridden at age 12 y; tracheostomy tube placed at 14 y | ─ | ─ | +  recognized by 5 y; gradual decline in childhood; can do most activities of daily living with some help at 34 y | 6/10 |
| **Slurred speech (HP:0001350)** | +  age of onset: 15 y; unable to talk at 36 y | +  age of onset: 12 y; unable to talk at 25 y | ─  staccato speech throughout life | +  age at onset: 10 y | ─  absent speech | ─  speech delay, about 30 words, sign language | ─ | 4/10  2 with absent speech |
| **Gait disturbance (HP:0001288)** | +  gait ataxia at 17 y; loss of ambulation at 20 y; spastic tetraparesis at 32 y; bedridden at 36 y | +  gait ataxia at 15 y; bedridden at 25 y | +  gait ataxia at 21 y;  balance problems with frequent falls; at 23.5 y: wheelchair bound, later with full assist required to transfer and walk steps | +  gait ataxia, bedridden at 12 y | ─  could walk with help at 4−5 y; after 14 y he could walk without help; at about 20 y he could not see and since then he needs help to walk | +  unsteady gait | ─ | 7/10 |
| **Abnormality of movement (HP:0100022)** | +  limb tremor at 36 y | +  limb and intention tremor; dysmetria at 25 y | not reported | +  dystonia at 8 y | not reported | +  myoclonus in early childhood; improved with levetiracetam | +  intermittent hand tremor, onset in childhood | 6/6 |
| **Seizures (HP:0001250)** | +  age at onset: 32 y | ─ | +  first seizure at 9 m; Lennox-Gastaut syndrome; stopped at 14 y when starting zoloft | +  first febrile episode at 2 y; myoclonic seizures | ─ | +  generalized tonic clonic seizures since 13 m; Lennox-Gastaut syndrome | +  infantile spasms at 4 m; many seizure types in childhood, initially intractable then well controlled by teens; admitted with multiple seizures at 34 y | 8/10  controlled on medication in 4 subjects, temporarily controlled for years in 1 subject |
| **EEG abnormality (HP:0002353)** | not done | not done | ─  normal at 25 y | +  right hemispheric epileptic discharges, clinically left focal seizure at 16 y | ─ | +  at 13 m; recurrent polyspike and wave discharges seen in a generalized distribution as bursts with a posterior predominance | +  at 28 y: disturbance of cerebral cortical function involving the right parietal-temporal-occipital area. Occasional focal low amplitude epileptiform abnormalities in right occipital area | 5/8 |
| **Behavioral abnormality (HP:0000708)** | ─ | ─ | +  obsessive compulsive disorder got worse at 14 y; zoloft started; impaired social awareness and lacked empathy for others | +  sleep disorder at 12-13 y; melatonin and clobazam started | +  motor stereotypy | ─ | +  focus issues in childhood.  psychosis episodes starting at 25 y;  cataplexy episode at 25 y; occasional night terrors | 5/10 |
| **CT brain abnormalities** | +  enlarged cisterna magna (at 17 y), diffuse cerebral and cerebellar atrophy, with compensatory  hydrocephalus (at  36 y) | not done | not done | not done | not done | not done | +  at 34 y: multiple ill-defined non-enhancing low-attenuation areas in both the cerebral hemispheres predominantly in the parietal regions. | 4/5 |
| **Brain MRI abnormalities** | not done | +  pineal cyst, *ex vacuo* dilatation of the ventricles | +  at 18 y: cerebral cortical atrophy; cerebral cortical atrophy stable at 22 y, 23 y, and 25 y | +  normal at 10 y; at 10 y and 6 m: cerebral, cerebellar, and brainstem atrophy | not available | +  at 18 m: prominent perivascular spaces in the periventricular white matter posteriorly, left more than the right; nonspecific white matter signal changes compatible with delayed myelination, dysmyelination, nonspecific leukodystrophy etc. | +  at 34 y: mild cerebellar atrophy and some periventricular and subcortical white matter hyper-intensities on T2 FLAIR | 7/8 |
| + |  |  |  |  |  |  |  |  |
| **Visual loss (HP:0000572)** | not reported | ─ | +  at 36 w; retinopathy of prematurity bilaterally, legally blind; right phthisis bulbi with metastastic bone formation (on pathology); s/p eye enucleation (at 15 y) | +  at 20 y  (family description) | +  at ~20 y operated for cataract; after three months he lost vision again | ─ | ─ | 5/9 |
| **Abnormal fundus morphology (HP:0001098)** | not reported | not reported | +  right eye retinal detachment, proliferative vitreoretinopathy, vitreous hemorrhage with secondary glaucoma; s/p cryotherapy and laser treatment at 8 y | not done | not done | ─ | not done | 3/5 |
| **Abnormal retinal morphology on macular OCT (HP:0030612)** | not reported | not reported | not done | not done | not done | not done | not done | 1/2 |
| **Hearing impairment (HP:0000365)** | no data | no data | ─  normal at 25 y | no data | no data | ─ | +  minor hearing issues, not tested recently | 3/6 |
| **Additional abnormalities** | | | | | | | | |
| **Flexion contracture (HP:0001371)** | +  at age 36 y: affected fingers | ─ | +  tendons in toes had to be cut at 10 y; in the last years, arms and legs affected | +  age at onset: 12 y;  affected fingers and toes | +  fingers and toes after age 4-5 y; tibial deviation of the 4th toe of the right foot since early ages | ─ | ─ | 7/10 |
| **Respiratory failure requiring assisted ventilation (HP:0004887)** | +  tracheostomy at 15 y, dependent on assisted ventilation at 36 y | +  tracheostomy at 15 y | ─ | +  tracheostomy at 14 y | ─ | ─ | ─ | 3/10 |
| **Abnormality of blood and blood-forming tissues (HP:0001871)** | not reported | +  idiopathic pancytopenia | +  thrombocytopenia | not reported | +  leukopenia | +  pancytopenia since 8 y (for >2 y); improving with eltrombopag (transfusion-dependency and neutropenia resolved, thrombocytopenia improving) | +  mild lymphopenia and intermittent mild thrombo-cytopenia | 5/5  2 with pancyto-penia |
| **Other features** | soft tissue growth in the maxillar region leading to maxillary hypertrophy; eye sockets deformity  with enlargement and lateralization of  eyeballs | congenital strabismus  and high myopia; soft tissue growth in the maxillar region leading to maxillary hypertrophy;  mild mitral valve  prolapse; joint stiffness of the right upper limb at age 25 y | born prematurely at 28 weeks gestation (515 g) (mother had pre-eclampsia), was born small; small for her age throughout life; hypoalgesia (she did seem to experience pain, particularly by end of life); protein, calcium and albumin low (started once she started to decline and progressively lowered overtime); CRP chronically elevated since age of 21 y | was playing in the school volleyball team before the age of 10 y; diabetes mellitus at 10 y; speech disorder and progressive disease after the age of 10 y | born at 39-40 weeks gestation by vaginal delivery; was hospitalized because of meconium aspiration/asphyxia; thenar and hypothenar atrophy of hands; ptosis of right eye; once operated for dental surgery (about teeth health); operated for tip-toe gait at ~2 y | microcephaly, slight right eye esotropia | somewhat prominent premaxillary region; family notices nystagmus when tired or stressed; small feet; episode of asystole followed by 3rd degree AV heart block during seizure at 34 y, requiring prophylactic pacemaker; normal echocardiogram at 34 y |  |

^a^ Only a total of ten subjects were included as genotyping of the *TBC1D2B* variant could not be done in subject 2 because of lack of DNA.

^b^ possibly drug induced.

+, present; ̶ , absent; CRP, C-reactive protein; CT, computerized tomography; m, months; MRI, magnetic resonance imaging; OCT, optical coherence tomography; VEP, visual evoked potential; w, weeks; y, years.

**Supplemental References**

1. Mencacci NE, Kamsteeg EJ, Nakashima K, R'Bibo L, Lynch DS, Balint B, et al. De Novo Mutations in PDE10A Cause Childhood-Onset Chorea with Bilateral Striatal Lesions. Am J Hum Genet. 2016;98(4):763-71.

2. Robinson JT, Thorvaldsdottir H, Winckler W, Guttman M, Lander ES, Getz G, et al. Integrative genomics viewer. Nat Biotechnol. 2011;29(1):24-6.

3. Kircher M, Witten DM, Jain P, O'Roak BJ, Cooper GM, Shendure J. A general framework for estimating the relative pathogenicity of human genetic variants. Nat Genet. 2014;46(3):310-5.

4. Ioannidis NM, Rothstein JH, Pejaver V, Middha S, McDonnell SK, Baheti S, et al. REVEL: An Ensemble Method for Predicting the Pathogenicity of Rare Missense Variants. American Journal of Human Genetics. 2016;99(4):877-85.

5. Jagadeesh KA, Wenger AM, Berger MJ, Guturu H, Stenson PD, Cooper DN, et al. M-CAP eliminates a majority of variants of uncertain significance in clinical exomes at high sensitivity. Nat Genet. 2016;48(12):1581-6.

6. Capriotti E, Altman RB, Bromberg Y. Collective judgment predicts disease-associated single nucleotide variants. BMC Genomics. 2013;14 Suppl 3:S2.

7. Coban-Akdemir Z, White JJ, Song X, Jhangiani SN, Fatih JM, Gambin T, et al. Identifying Genes Whose Mutant Transcripts Cause Dominant Disease Traits by Potential Gain-of-Function Alleles. Am J Hum Genet. 2018;103(2):171-87.

8. Harms FL, Parthasarathy P, Zorndt D, Alawi M, Fuchs S, Halliday BJ, et al. Biallelic loss-of-function variants in TBC1D2B cause a neurodevelopmental disorder with seizures and gingival overgrowth. Hum Mutat. 2020;41(9):1645-61.

9. Correia-Costa GR, de Leeuw N, Pfundt R, Sgardioli IC, Dos Santos AP, de Lima Santos M, et al. Biallelic frameshift variant in the TBC1D2B gene in two siblings with progressive gingival overgrowth, fibrous dysplasia of face, and mental deterioration. Clin Genet. 2022;102(6):537-42.
